# Titanium Versus Bioabsorbable Magnesium Headless Compression Screw Fixation for Tibial Tubercle Osteotomy

**DOI:** 10.3390/jfb16110404

**Published:** 2025-10-29

**Authors:** Mehmet Baris Ertan, Oguzhan Uslu, Firat Dogruoz, Omer Faruk Celik, Ozkan Kose

**Affiliations:** 1 Orthopedics and Traumatology Clinic, Medikum Private Hospital, Antalya 07350, Turkey; mehmetbarisertan@gmail.com; 2 Department of Orthopedics and Traumatology, Antalya Training and Research Hospital, University of Health Sciences, Antalya 07010, Turkey; oguzhannuslu@gmail.com (O.U.); firatdogruoz@hotmail.com (F.D.); comer_2005@hotmail.com (O.F.C.); 3 Antalya Training and Research Hospital, Varlık Neighborhood, Kazım Karabekir Street, Antalya 07100, Turkey

**Keywords:** tibial tubercle osteotomy, fulkerson osteotomy, bioabsorbable magnesium screw, titanium headless screw, implant removal, patellofemoral instability, osteotomy fixation

## Abstract

Purpose: This retrospective study aimed to compare the clinical and radiological outcomes of Fulkerson tibial tubercle osteotomy (TTO) fixed with either bioabsorbable magnesium (Mg) or titanium (Ti) headless compression screws, particularly focusing on the need for implant removal. Materials and Methods: 29 patients (19 Ti, 10 Mg screws) who underwent TTO between 2013 and 2023 were included. The primary outcome was implant removal; secondary outcomes included Kujala and Lysholm scores, complication rates, and radiographic union. Rehabilitation protocols were standardized, but early weight-bearing was delayed in the Mg group to mitigate potential implant degradation effects. Results: Both groups demonstrated significant improvements in Kujala and Lysholm Knee scores postoperatively, with no statistically significant differences between the groups. No cases of implant removal, fixation failure, nonunion, or significant range of motion loss were observed. Radiographically, persistent remnants of Mg screws were detected even after more than five years, raising concerns about incomplete bioabsorption. The Ti screws maintained mechanical stability without evidence of loss of fixation or the need for revision. Patient satisfaction and cosmetic outcomes were similar. Conclusion: The use of bioabsorbable Mg screws in TTO did not confer additional clinical or radiological benefits compared to headless Ti screws. Given the higher cost, the incomplete resorption observed at long-term follow-up, and the absence of an implant removal requirement in either group, the routine use of Mg screws cannot be recommended. Ti headless compression screws offer a cost-effective, reliable fixation method, achieving stable osteotomy healing without the need for secondary surgery.

## 1. Introduction

Tibial tubercle osteotomy (TTO) is a commonly performed procedure for managing patellofemoral instability and malalignment. Among various TTO techniques, the Fulkerson osteotomy, which allows for anteromedialization of the tibial tubercle, has been widely adopted due to its ability to address both patellar maltracking and increased tibial tubercle–trochlear groove (TT–TG) distance [1,2,3]. Stable fixation of the osteotomy fragment is critical to ensure union, facilitate early mobilization, and prevent complications such as displacement or nonunion. Traditionally, fixation has been achieved using metallic screws [4,5,6]. However, irritation from screw head prominence and subsequent implant removal remain common postoperative concerns [7,8]. In a systematic review of 787 tibial tubercle osteotomies, symptomatic hardware removal was required in 49.0% of cases using the Fulkerson technique [9]. Some authors routinely recommend implant removal after tibial tubercle transfer to prevent kneeling discomfort and avoid potential screw breakage at a later stage [10].

To reduce the need for secondary surgery, bioabsorbable magnesium (Mg) screws have emerged as a promising alternative to traditional metallic fixation implants [11,12]. These implants offer the theoretical advantage of sufficient mechanical strength combined with gradual resorption, thereby eliminating hardware-related complications and the necessity for implant removal. In contrast to traditional metallic implants, which often require removal due to irritation, pain, or risk of breakage or failure, magnesium-based screws are designed to degrade in vivo over time without eliciting adverse tissue reactions [13,14,15,16,17,18,19]. Bioabsorbable alternatives may represent a promising strategy to reduce patient morbidity, surgical burden, and overall healthcare costs while maintaining clinical efficacy.

The short-term clinical and radiological outcomes of patients who underwent TTO fixed with bioabsorbable Mg headless compression screws were previously reported [20]. Although the results were promising, that study lacked a comparative group utilizing conventional implants. In the present study, the clinical and radiological outcomes of patients undergoing TTO with either bioabsorbable Mg or titanium (Ti) headless compression screws were compared. By utilizing a comparable cohort of patients treated with Ti implants, the efficacy and safety of bioabsorbable Mg screws as an alternative fixation method in TTO were evaluated. The study was therefore designed to compare the clinical and radiological outcomes between bioabsorbable Mg and Ti screw fixation methods in tibial tubercle osteotomy (TTO). In particular, an evaluation was conducted to determine whether bioabsorbable Mg screws would provide an advantage regarding the need for implant removal. The study hypothesized that the necessity for implant removal would be eliminated through the use of Ti headless compression screws, similar to bioabsorbable screws.

## 2. Materials and Methods

### 2.1. Patients, Study Design, and Sample Size Calculation

**Figure 1:**
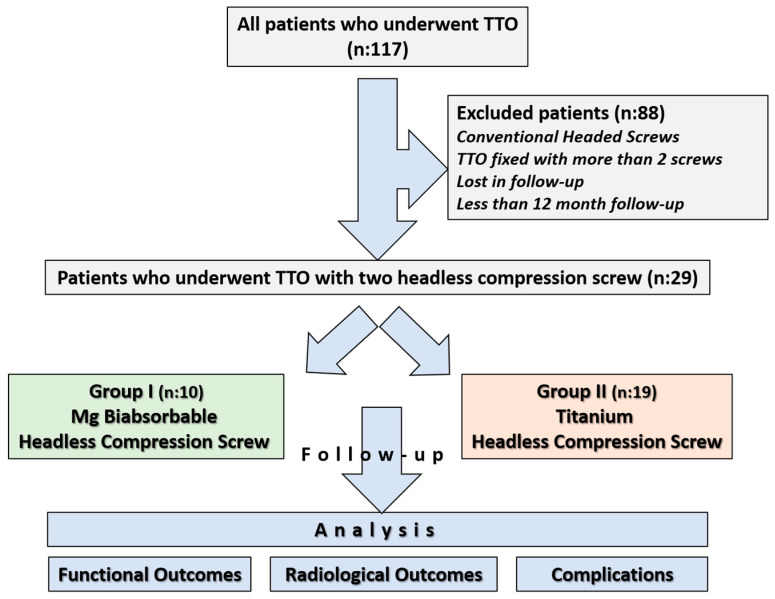
Flowchart illustrating patient selection and grouping.

Ethical approval for the study was obtained from the institutional review board prior to its initiation, and informed consent was obtained from all participants (Approval date and issue: 3 September 2025). The study was conducted in accordance with the principles outlined in the Declaration of Helsinki. All patients who underwent tibial tubercle osteotomy between 2013 and 2023 were retrospectively reviewed using the institutional database. 117 patients who underwent Fulkerson osteotomy during this period were identified. In our department, Fulkerson osteotomy is generally stabilized with either two or three screws, depending on the surgeon’s preference. Among the identified patients, 10 who underwent fixation with Mg bioabsorbable headless compression screws, each treated with two screws, were selected for the study group. To avoid potential bias associated with screw number and design, and to ensure group homogeneity, the Ti group was likewise restricted to patients fixed with two headless compression screws. Thus, a study cohort was constituted in which both the Ti and Mg groups consisted exclusively of patients fixed with two headless compression screws (Two Mg screws versus two Ti screws). The exclusion criteria were as follows: use of conventional headed screws, use of more than two screws for fixation, follow-up duration of less than one year, incomplete clinical or radiological data, insufficient documentation, or loss to follow-up. Since the Mg group consisted of only 10 patients, it was necessary to confirm whether the available sample size would be sufficient to draw reliable conclusions. The sample size calculation was conducted to determine the required sample size for the comparative group using the G*Power software (version 3.1.9.7) [21]. The primary outcome was the comparison of implant removal rates between two different screw types: bioabsorbable and metallic. Based on previously published data, the implant removal rate was estimated to be 0% for bioabsorbable screws and approximately 49% for metallic screws used in Fulkerson TTO [9,20]. Given this expected substantial difference, a one-sided test was employed with a significance level (α) of 0.05 and a statistical power (1 − β) of 80%. Due to the limited number of patients in the bioabsorbable screw group, the allocation ratio was set at 2:1 in favor of the metallic screw group to enhance the statistical power. Under these assumptions, a minimum total of 20 patients (7 in the bioabsorbable screw group and 13 in the metallic screw group) was required to achieve sufficient power. After applying the exclusion criteria, a total of 29 patients were included in the study: 10 patients in the magnesium screw (Mg) group and 19 patients in the titanium screw (Ti) group (Figure 1).

### 2.2. Surgical Technique and Implants

All procedures were performed by the same surgical team under either spinal or general anesthesia, using a proximal thigh tourniquet. A longitudinal incision approximately 8–10 cm in length was made along the tibial crest at the level of the tibial tubercle. The borders of the patellar tendon were first identified, and the anterolateral compartment muscles were retracted laterally. An osteotomy was planned, beginning approximately 6–7 cm distal to the insertion of the patellar tendon. Two parallel Kirschner wires were inserted from medial to lateral at approximately a 45° angle to guide the osteotomy line. Using a motorized oscillating saw, the osteotomy was performed distally with a sloped configuration [22]. In cases where distalization was not required, care was taken to preserve the periosteum at the distal hinge. The proximal part of the osteotomy was completed with an osteotome. The osteotomized fragment was mobilized to create a “greenstick” fracture at the distal hinge, then medialized approximately 1–1.5 cm proximally, depending on the desired correction. Once the desired medialization was achieved, temporary fixation was performed with a single K-wire. Definitive fixation was then achieved using two screws inserted at positions dividing the osteotomy length into three equal segments. The screws were directed toward the posteromedial cortex and inserted perpendicular to the osteotomy surface, ensuring bicortical purchase in all cases.

**Figure 2:**
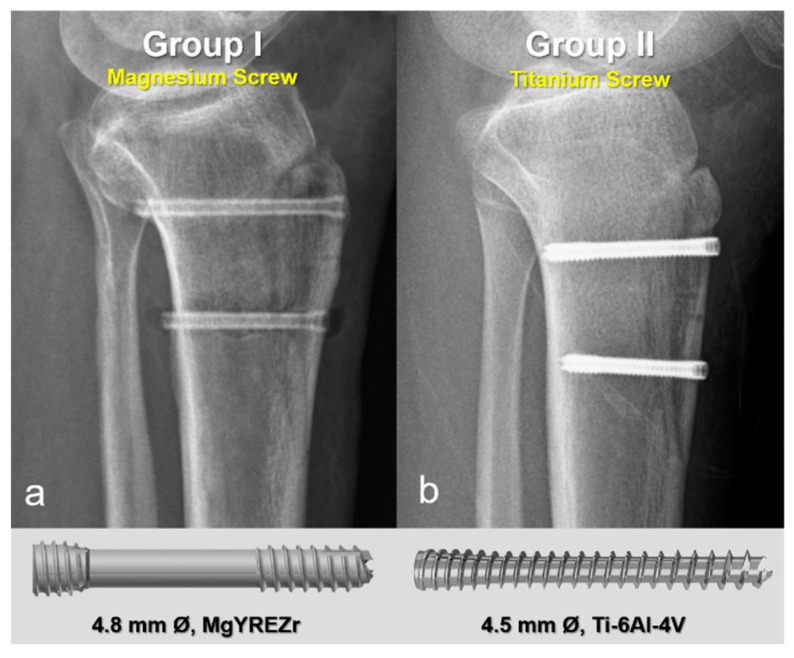
Representative postoperative radiographs and screw models used in each group. (**a**) Magnesium bioabsorbable screw (4.8 mm Ø, MAGNEZIX^®^ CSc, Syntellix AG, Hanover, Germany), featuring a headless, cannulated, Herbert-type design with variable pitch threading for self-compression and complete subcortical embedding. (**b**) Titanium headless compression screw (4.5 mm Ø), with a conical, cannulated, variable pitch design.

In the first group, fixation was performed using 4.8 mm diameter magnesium bioabsorbable headless compression screws (4.8 mm Ø, MAGNEZIX^®^ CSs, Syntellix AG, Hanover, Germany). These screws are headless, Herbert-type implants featuring a variable pitch thread design that enables self-compression during insertion, thereby promoting stable fixation and optimal bone healing. The screws are also cannulated, allowing for precise placement over a guide wire, and are fully embedded below the cortical surface to minimize soft tissue irritation. In the second group, 4.5 mm titanium headless compression screws with similar characteristics were used. These screws are likewise headless, cannulated, and variable pitch, functioning on the same biomechanical principles. Their design is comparable to Acutrak screws, incorporating a tapered core, aggressive thread profile, and conical geometry to provide substantial bone purchase and secure fixation, particularly in osteotomies and metaphyseal regions (Figure 2). All screws in both groups were countersunk to ensure they remained flush with the cortical bone surface, preventing implant prominence and soft tissue complications.

Except for one patient, all included cases underwent concomitant standard medial patellofemoral ligament reconstruction (MPFLR). Depending on the surgeon’s preference, either single- or double-bundle MPFLR was performed. Femoral and patellar tunnel placements were guided by fluoroscopy, targeting the anatomical insertion sites as described by Schöttle et al. [23]. In 18 cases, autografts (gracilis or semitendinosus tendons) were harvested using the same incision. In one patient, the anterior half of the peroneus longus tendon (AHPLT) was used as a graft. Closure was performed in layers, and a single closed-suction drain was placed. In cases of associated intra-articular patellar fractures, fixation was performed if the fragment was adequate (>1 cm); otherwise, small or comminuted fragments were excised. The drain was removed on the first postoperative day.

### 2.3. Postoperative Rehabilitation and Follow-Up

Postoperative rehabilitation protocols were standardized for all patients, with certain variations based on the type of screw used. Patients in the Mg group were mobilized with crutches, bearing no weight for the first two weeks. In contrast, those in the Ti group were permitted full weight-bearing starting from the first postoperative day. In both groups, early rehabilitation included controlled knee range-of-motion exercises limited to 0–90° of flexion, and quadriceps strengthening exercises were initiated with the support of a hinged knee brace. During the second and third postoperative weeks, patients were encouraged to bear full weight while wearing a locked knee brace in full extension. Patellofemoral mobilization exercises were also introduced, specifically in the superior, inferior, and medial directions, while lateral mobilization was avoided. By the end of the fourth week, patients were allowed to discontinue use of the brace, resume full weight-bearing, and perform knee range-of-motion exercises up to 120–140° of flexion as tolerated [24]. Return to sports or strenuous activities was allowed after complete union and consolidation of the osteotomy, with a negative apprehension test and regaining full range of motion (ROM) and muscle strength, typically after 4 to 6 months.

### 2.4. Radiological Evaluation

All patients underwent a standardized preoperative radiological evaluation, including direct radiographs, magnetic resonance imaging (MRI), computed tomography (CT), and full-length lower limb alignment radiographs. Trochlear dysplasia was assessed using the Dejour classification, based on CT or MRI scans. Patellar height was evaluated using the Caton-Deschamps index, and a value greater than 1.2 was classified as patella alta. Measurements of tibial tubercle–trochlear groove (TT–TG) distance, patellar tilt, and lower limb alignment were also performed. Following the osteotomy, bone healing was monitored through serial radiographs obtained at different intervals. Radiographic union was defined as the disappearance of the osteotomy line on imaging and the absence of local tenderness upon palpation. Due to the retrospective nature of the study and variable follow-up intervals between patients, the exact time to union could not be reliably determined. However, the presence or absence of union was confirmed on the final follow-up radiographs, which were obtained at least 12 months postoperatively. Radiographic union was assessed simultaneously by the senior author and the corresponding author, and the final judgment was made by consensus.

### 2.5. Assessment of Functional Outcomes and Complications

At the final follow-up, all patients underwent a comprehensive physical examination, including assessments of musculoskeletal and neurovascular function. Knee flexion and extension ranges were evaluated bilaterally and compared to the contralateral side. Muscle strength was assessed using manual muscle testing. During the knee examination, the presence of a J-sign and patellar apprehension was also evaluated. Patient-reported outcome measures included the Kujala Anterior Knee Pain Scale [25] and the Lysholm Knee Scoring Scale [26]. Additionally, patient satisfaction with the overall treatment process and outcome was assessed using a numeric rating scale ranging from 0 to 10, with higher scores indicating greater satisfaction. The patients rated the cosmetic appearance of the incision scars on a scale from 0 to 10 [27]. All complications throughout the treatment course were documented and recorded, including intraoperative, immediate postoperative, and long-term follow-up periods. Specific complications evaluated included wound healing problems, infection, nonunion, implant failure, revision surgery, painful implant, and implant removal.

### 2.6. Statistical Analysis

Continuous variables were expressed as mean ± standard deviation (SD) or median with range, depending on the distribution. Categorical variables were presented as frequencies and percentages. The normality of data distribution was assessed using the Shapiro–Wilk test. For continuous variables, comparisons between the magnesium (Mg) and titanium (Ti) groups were performed using the independent samples *t*-test or the Mann–Whitney U test, as appropriate. Categorical variables were compared using the chi-square test. For pre- and postoperative comparisons within each group, the Wilcoxon signed-rank test was used for non-normally distributed variables. A *p*-value of less than 0.05 was considered statistically significant. Statistical tests used for each comparison are indicated in the corresponding tables.

## 3. Results

A total of 29 patients who met the inclusion criteria were analyzed, with 10 patients in the magnesium (Mg) group and 19 patients in the titanium (Ti) group. Baseline demographic and clinical characteristics were comparable between the groups. No statistically significant differences were observed in age, sex distribution, BMI, ASA scores, TT–TG distance, patellar tilt, or type of trochlear dysplasia. Although the Caton-Deschamps index was significantly lower in the Mg group (*p* = 0.008), the proportion of patients with patella alta was similar (Table 1).

Perioperative characteristics, including the rate of concomitant procedures, graft choices, type of MPFL reconstruction, anesthesia method, and hospital stay, were similar between groups. Although the mean operative time and osteotomy length appeared slightly greater in the Mg group, the differences did not reach statistical significance (Table 2).

Radiological and clinical follow-up durations were significantly longer in the Mg group, reflecting the retrospective nature of this cohort and the earlier use of Mg implants (*p* = 0.001 for both). Functional outcomes, assessed by Kujala and Lysholm Knee Scores, improved significantly from preoperative to postoperative follow-up in both groups (*p* < 0.01 for within-group comparisons), with no statistically significant difference in final scores between the Mg and Ti groups. Likewise, patient-reported satisfaction scores, including both overall and cosmetic outcomes, were high in both groups and statistically similar (Table 3).

**Figure 3:**
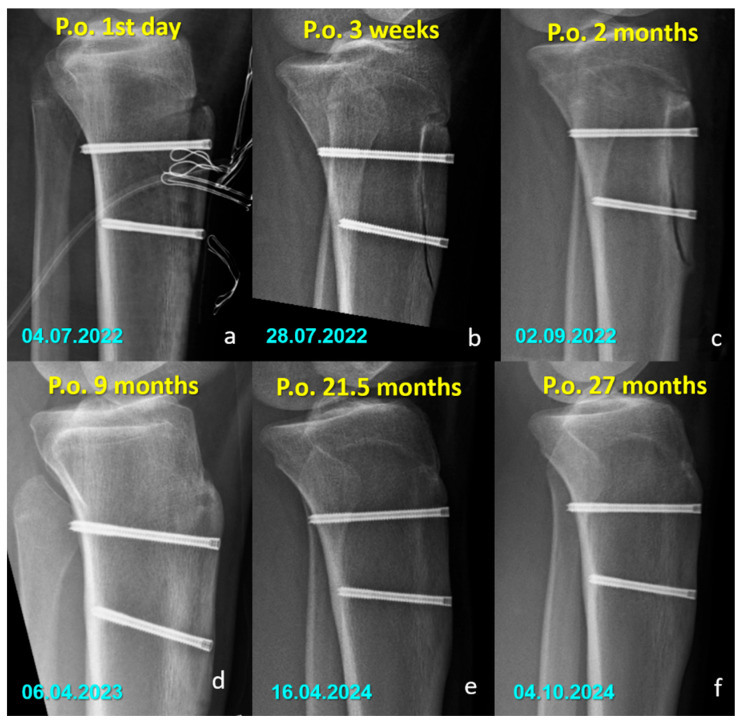
Serial lateral radiographs (**a**–**f**) showing the postoperative course of a patient treated with titanium compression screws following Fulkerson osteotomy.

**Figure 4:**
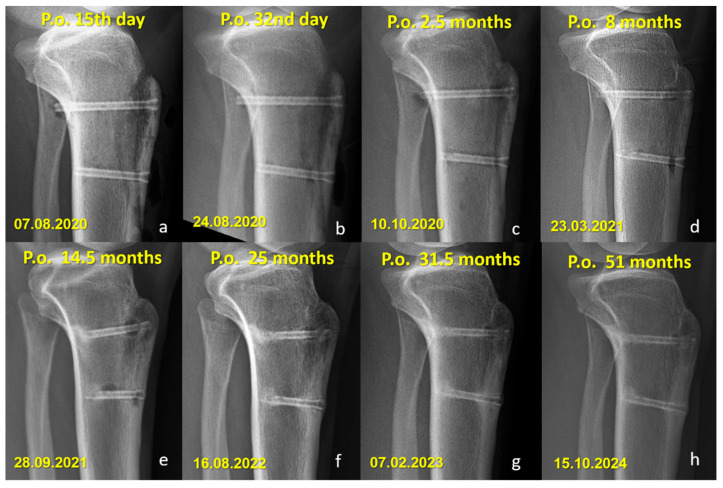
Serial lateral radiographs showing long-term follow-up of a patient who underwent Fulkerson osteotomy fixed with bioabsorbable Mg screws. (**a**–**h**) Progressive time points from 15 days to 51 months postoperatively demonstrate bone healing, continued remodeling, and gradual resorption of the magnesium screws.

There were no cases of implant removal, fixation failure, nonunion, or significant deficits in range of motion or strength in either group. Minor sensory disturbances (hypoesthesia) were observed in both groups (40% in the Mg group and 52.6% in the Ti group, *p* = 0.400), but without statistical significance. These findings were considered procedure-related, most likely due to osteotomy dissection or graft harvesting, rather than being implant-specific. Importantly, none of the patients required revision surgery, and no implant-related complications were observed during the follow-up period. All osteotomies united without displacement (Figure 3 and Figure 4).

### Radiographic Findings in the Mg Screw Group

**Figure 5:**
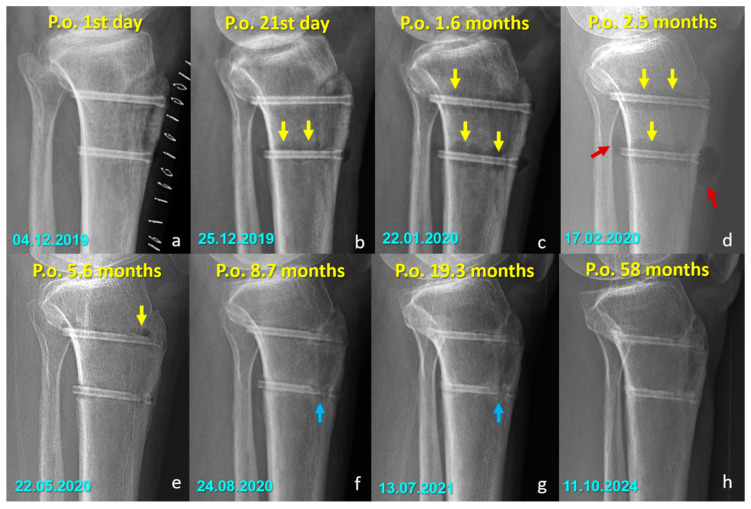
Serial radiographs demonstrating the temporal changes associated with magnesium screw degradation following Fulkerson osteotomy. (**a**–**d**) Early postoperative images show radiolucent zones and intramedullary or soft tissue gas shadows (yellow arrows), indicating hydrogen release during magnesium corrosion. In (**d**), gas accumulation is evident in the adjacent soft tissues (red arrows). (**e**) By 5.6 months, early signs of material degradation appear. (**f**–**h**) In the later stages, blue arrows highlight the loss of screw integrity, particularly along the osteotomy line, reflecting ongoing degradation and fragmentation of the implant. At 58 months (**h**), radiodense remnants of the screw are still visible, indicating that complete bioabsorption was not achieved in this case. (Arrow color code, yellow = radiolucent zones/gas shadows, red = soft tissue gas accumulation, blue = loss of screw integrity).

**Figure 6:**
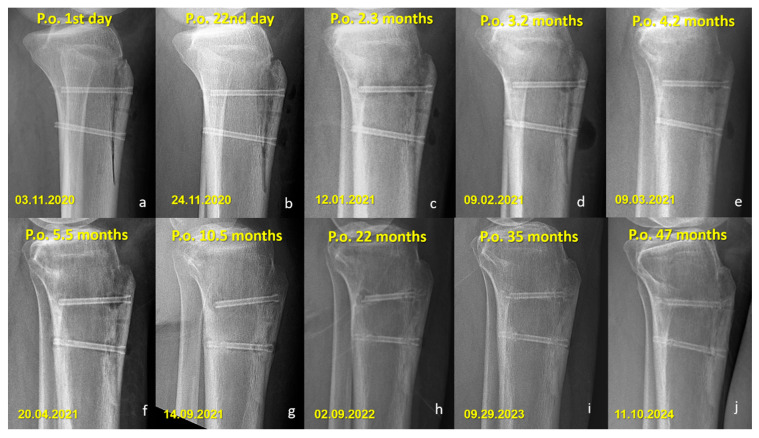
Sequential lateral radiographs of a patient who underwent Fulkerson osteotomy fixed with bioabsorbable Mg screws, showing the typical radiographic evolution over a 47-month follow-up period. (**a**–**e**) In the early postoperative period, radiolucent areas around the screws are visible, consistent with hydrogen gas release during magnesium degradation. (**f**–**h**) Progressive changes indicate gradual absorption of the screws, with a noticeable decrease in screw density and partial loss of structural integrity over time. (**i**,**j**) Despite ongoing degradation, remnants of the screw material remain visible at 35 and 47 months, indicating incomplete resorption. No complications such as osteolysis, displacement, or nonunion are observed throughout the follow-up.

Distinct radiographic features were observed in the Mg screw group, attributed to the bioabsorption characteristics of the implant. In the early postoperative period, radiolucent zones and gas shadows were frequently noted around the screws. These findings are consistent with the release of hydrogen gas during the degradation process of magnesium. In some cases, gas accumulation extended into adjacent soft tissues. Over time, the amount of gas gradually decreased, suggesting a deceleration of the corrosion process and partial absorption of the released hydrogen. In the later stages, partial degradation of the screw, particularly along the osteotomy line, was observed in several cases, indicating progressive material absorption. However, full resorption of magnesium screws with restoration of normal bone architecture was not observed in any patient. Radiographically dense remnants persisted in all cases (100%), raising concerns about the completeness of bioabsorption. Notably, 9 of 10 patients had radiological follow-up longer than three years, all of whom demonstrated persistent screw remnants (Figure 5 and Figure 6).

## 4. Discussion

In this study, the clinical and radiological outcomes of Fulkerson osteotomy fixed with either bioabsorbable Mg headless compression screws or Ti headless compression screws were evaluated. It was demonstrated that the use of bioabsorbable screws did not confer additional clinical or radiological advantages compared to titanium screws. Given the absence of differences in functional outcomes and implant-related complications between the two groups, the routine use of more expensive bioabsorbable magnesium screws in this setting is not recommended. The findings of the present study suggest that headless titanium screws provide a cost-effective and reliable fixation method, eliminating the need for subsequent implant removal in tibial tubercle osteotomy (TTO).

The short-term outcomes of patients treated with Mg screws had previously been reported [20]. The current investigation extends those findings by demonstrating that the favorable functional outcomes were maintained at long-term follow-up, with no observed deterioration. Nevertheless, complete resorption of the magnesium screws, as initially anticipated, was not achieved even after more than five years postoperatively. Persistent radiodense remnants were identified on imaging, indicating that the in vivo corrosion process of the magnesium implants likely ceases after a certain period. While the exact nature of the tissue formed at the site of maximal screw degradation remains uncertain, radiological findings suggest that it is unlikely to represent connective tissue, as this would typically result in a completely lytic appearance. Instead, the persistence of dense radiographic features supports the hypothesis that either of the screws were replaced by cortical-like bone tissue. Alternatively, a superficial dense layer might have developed, which could have prevented further interaction between the implant and body fluids, thereby stopping the degradation process. The similarity between the early and long-term radiological appearances of the screws (depicted in Figure 4, Figure 5 and Figure 6) further reinforces this assumption. Although these interpretations remain speculative, they provide valuable insight into the potential long-term behavior of magnesium implants. Ideally, such observations should be validated through long-term in vivo animal studies and detailed histopathological analyses to precisely characterize the tissue response and degradation dynamics of magnesium screws.

The main theoretical advantage of magnesium implants lies in their ability to eliminate the need for implant removal. However, the initial cost of bioabsorbable magnesium screws is substantially higher than that of titanium screws [28]. Based on the findings of this study, since implant removal was not required in either group, the use of titanium headless screws appears to offer an equally effective yet more economical alternative. Consequently, the routine use of bioabsorbable magnesium screws in Fulkerson osteotomy cannot be justified based on the present results. Another critical concern regarding Mg implants is the potential loss of biomechanical strength over time due to in vivo corrosion [29,30]. Early weight-bearing was initially restricted in the magnesium group to mitigate the risk of fixation failure, and a more gradual rehabilitation protocol was subsequently implemented. In contrast, titanium screws provided immediate and sustained mechanical stability, allowing for early mobilization without compromising fixation integrity. These differences in rehabilitation protocols should be considered when selecting fixation materials for TTO procedures.

An important consideration regarding tibial tubercle osteotomy (TTO) is the necessity for implant removal due to symptomatic hardware. Previous studies have reported highly variable hardware removal rates, with Payne et al. noting that removal rates reached up to 49% in their systematic review [9]. The presence of prominent screw heads has been identified as a major contributor to postoperative discomfort, particularly during activities such as kneeling, leading to secondary surgeries. Lehane et al., in a recent large cohort study involving 476 TTOs, demonstrated that painful hardware requiring removal was the most common major complication, with an overall hardware removal rate of 6.5%. Importantly, they found that hardware removal was significantly more frequent with headed screws compared to headless screws (13.2% vs. 1.7%, *p* < 0.001) [31]. These findings emphasize the critical role of implant design in minimizing soft tissue irritation and subsequent reoperation rates. In our study, the exclusive use of fully embedded headless compression screws resulted in no cases of implant removal, despite long-term follow-up. This outcome strongly suggests that headless screw designs can substantially mitigate hardware-related complications, reducing the need for secondary procedures without necessitating the additional costs and bioabsorption concerns associated with biodegradable implants. Consequently, the use of titanium headless compression screws appears to provide a cost-effective and reliable solution for TTO fixation, offering the mechanical stability necessary for osteotomy healing while minimizing symptomatic hardware complications effectively.

Bioabsorbable magnesium implants are generally more expensive than conventional metallic devices, resulting in higher upfront surgical costs compared to titanium or steel screws. Nevertheless, several cost-analysis studies have argued that, when secondary operations for implant removal are taken into account, bioabsorbable implants may ultimately be more economical. For example, Juutilainen et al. analyzed 140 patients treated for ankle fractures (92 with bioabsorbable and 48 with metallic screws) and demonstrated that, after considering both primary surgery and subsequent implant removal, bioabsorbable screws could be more cost-effective [32]. Similarly, Böstman et al. reported that a financial advantage could only be achieved if the implant removal rate exceeded 21% in unimalleolar fractures [33]. In another investigation, Klauser estimated that assuming an 8% removal rate in hallux valgus osteotomy fixation, the routine use of bioabsorbable magnesium screws across Germany could result in savings of approximately €9 million [34]. Based on such findings, the literature often suggests that the potential cost-effectiveness of magnesium screws is largely dependent on the frequency of implant removal in conventional screw fixation. In contrast, in our study, no patient in either group required implant removal. Since magnesium screws are considerably more expensive than titanium screws, and the theoretical cost-saving advantage linked to secondary procedures did not apply, the overall initial implant cost was actually higher in the magnesium group.

This study constitutes the first comparative analysis of bioabsorbable magnesium and titanium headless compression screws in Fulkerson osteotomy, offering valuable insights into their relative clinical and radiological performance. A key strength of the study is the use of a standardized surgical technique across all cases, which helps minimize potential confounding factors. However, certain limitations should be acknowledged. First, there was a difference in postoperative rehabilitation protocols between the groups. Because magnesium screws have lower initial mechanical strength and progressively lose stability due to degradation, weight-bearing was deliberately delayed in the Mg group to reduce the risk of fixation failure. In contrast, early full weight-bearing was permitted in the Ti group because of the consistent stability provided by titanium screws. Although this difference may have influenced early functional recovery, the considerably longer mean follow-up in the Mg group and the absence of significant differences in Kujala and Lysholm scores at final follow-up suggest that these protocol variations did not confound the long-term outcomes. Second, the follow-up durations between groups were not equal. Magnesium screws were used in earlier cases, resulting in significantly longer follow-up periods compared with the titanium cohort. Although the mean follow-up of 20 months in the Ti group is sufficient to detect implant-related complications such as irritation or removal, this imbalance may still restrict the comparability of very long-term findings. Third, the retrospective design and relatively small sample size, particularly in the Mg group, may limit the generalizability of the results. The sample size was estimated a priori with power analysis and deemed sufficient to support the study’s conclusions. However, this analysis was based on previously reported removal rates for metallic screws of up to 49%. In our cohort, no implant removal occurred in either group, most likely due to the exclusive use of headless screw designs. This discrepancy reduces the direct applicability of the initial power assumptions and should therefore be interpreted with caution. Finally, a quantitative assessment of the degradation process was not possible. The retrospective design and irregular radiographic follow-up intervals prevented precise measurement of resorption rates. Moreover, invasive techniques to monitor in vivo degradation are not ethically feasible in human subjects. Future prospective studies with standardized imaging protocols or long-term animal models incorporating histopathological evaluation may help clarify the degradation dynamics of magnesium screws more accurately.

## 5. Conclusions

In the fixation of Fulkerson osteotomy, both bioabsorbable magnesium and titanium headless compression screws yielded comparable clinical and radiological outcomes. The utilization of bioabsorbable magnesium screws did not yield any additional functional benefits, and neither group exhibited a requirement for implant removal. Despite the theoretical advantage of gradual resorption, magnesium screws were not completely absorbed even at long-term follow-up, and concerns regarding incomplete degradation persist. The findings of this study indicate that, given the higher cost of magnesium screws and the absence of clinical superiority, their routine use in tibial tubercle osteotomy procedures is not supported by the present findings. The use of headless titanium screws has been demonstrated to provide a cost-effective, stable, and reliable fixation method, thereby obviating the need for secondary implant removal without compromising outcomes.

## Figures and Tables

**Table 1:** Comparison of demographic and clinical characteristics of the patients in each group.

Variables	Mg Group (*n*:10)	Ti Group (*n*:19)	*p*-Value
Age (years ± SD)	23.4 ± 9.2	19.7 ± 5.8	0.247 ^1^
Median (Range)	20 (15–45)	17 (12–30)	
Sex (*n*, %)			0.283 ^2^
Male	5 (50%)	6 (32.6%)	
Female	5 (50%)	13 (68.4%)	
Side (*n*, %)			0.636 ^2^
Right	3 (30%)	6 (32.6%)	
Left	7 (70%)	13 (68.4%)	
Weight (kg ± SD)	171.9 ± 9.7	164.5 ± 9.5	0.060 ^3^
Height (cm ± SD)	72.4 ± 14.6	62.6 ± 13.2	0.082 ^3^
BMI (kg/m^2^ ± SD)	24.5 ± 5.2	23.1 ± 4.0	0.430 ^3^
ASA Score			0.364 ^2^
ASA I	6 (60%)	14 (73.7%)	
ASA II	4 (40%)	5 (26.3%)	
Tobacco use (*n*, %)	2 (20%)	3 (15.8%)	0.576 ^2^
Diabetes Mellitus	1 (10%)	0 (0%)	0.345 ^2^
Acute vs. Recurrent (*n*, %)			0.385 ^2^
Acute	5 (50%)	7 (36.8%)	
Recurrent	5 (50%)	12 (63.2%)	
TT-TG (mm ± SD)	20.4 ± 3.2	20.4 ± 3.2	0.558 ^1^
Patellar Tilt (° ± SD)	27.9 ± 8.7	27.9 ± 10.0	0.991 ^3^
Caton-Deschamps Index (ratio ± SD)	1.02 ± 0.1	1.16 ± 0.1	**0.008 ^3^**
Patella Alta (*n*, %)	1 (10%)	3 (15.8%)	0.571 ^2^
Dejour Classification (*n*, %)			0.291 ^2^
Type A	5 (50%)	8 (42.1%)	
Type B	5 (50%)	7 (36.8%)	
Type C	0	3 (15.8%)	
Type D	0	1 (5.3%)	
Lower Limb Alignment			0.555 ^2^
Normal	6 (60%)	15 (78.9%)	
Varus	2 (20%)	2 (10.5%)	
Valgus	2 (20%)	2 (10.5%)	

^1^ Mann–Whitney U-test, ^2^ Chi-square test, ^3^ Student *t*-test. Abbreviations: Mg: Magnesium, Ti: Titanium, SD: Standard deviation, BMI: Body mass index, ASA: American Society of Anesthesiologists, TT–TG: Tibial tubercle trochlear groove distance. Bold *p*-values are statistically significant.

**Table 2:** Comparison of perioperative characteristics.

Variables	Mg Group (*n*:10)	Ti Group (*n*:19)	*p*-Value
Osteotomy Length (cm ± SD)	8.9 ± 1.3	7.1 ± 1.8	0.179 ^1^
Median (range)	7.8 (6.6–11.1)	7.4 (6.4–9.1)	
Concomitant Procedures			
MPFLR	9 (90%)	19 (100%)	0.345 ^2^
Lateral Capsular Lengthening	0 (0%)	2 (10.5%)	0.421 ^2^
OCF Fixation	2 (20%)	2 (10.5)	0.429 ^2^
OCF Removal	1 (10%)	1 (5.3%)	0.579 ^2^
Graft option for MPFLR			0.655 ^2^
Hamstring tendons	9 (100%)	18 (94.7%)	
AHPLT	0 (0%)	1 (5.3%)	
Single vs. Double Bundle MPLR			0.548 ^2^
Single Bundle	8 (88.9%)	18 (94.7%)	
Double Bundle	1 (11.1%)	1 (5.3%)	
Duration of Operation (min ± SD)	101.5 ± 14.3	93.1 ± 11.0	0.093 ^3^
Type of Anesthesia			0.429 ^2^
Spinal	8 (80%)	17 (89.5%)	
General	2 (20%)	2 (10.5%)	
Length of Stay (days ± SD)	1.6 ± 0.6	1.3 ± 0.6	0.377 ^1^
Median (range)	1.5 (1–3)	1 (1–3)	

^1^ Mann–Whitney U-test, ^2^ Chi-square test, ^3^ Student *t*-test. Abbreviations: Mg, Magnesium; Ti, Titanium; SD, Standard deviation; MPFLR, Medial Patellofemoral Ligament Reconstruction; AHPLT, Anterior Half of Peroneus Longus Tendon; OCF, Osteochondral Fragment.

**Table 3:** Comparison of functional outcomes.

Variables	Mg Group (*n*:10)	Ti Group (*n*:19)	*p*-Value
Radiological Follow-up (months ± SD)	47.9 ± 14.8	20.1 ± 6.1	0.001 ^1^
Median (range)	50 (12–62)	19 (12–31)	
Clinical Follow-up (months ± SD)	56.7 ± 4.4	25.8 ± 6.7	0.001 ^1^
Median (range)	58(47–62)	25 (13–36)	
Kujala Score (point ± SD)			
Preoperative	47.9 ± 18.9	53.7 ± 22.4	0.456 ^1^
Postoperative	93.3 ± 9.4	94.8 ± 8.2	0.668 ^1^
*p*-value	0.005 ^2^	0.001 ^2^	
LKS (point ± SD)			
Preoperative	55.9 ± 22.5	58.7 ± 17.4	0.946 ^1^
Postoperative	95.0 ± 5.1	96.4 ± 4.6	0.484 ^1^
*p*-value	0.005 ^2^	0.001 ^2^	
Overall Satisfaction (point ± SD)	9.6 ± 0.9	9.2 ± 0.9	0.266 ^1^
Cosmetic Satisfaction (point ± SD)	8.2 ± 1.9	8.0 ± 1.8	0.735 ^1^
Postoperative Extension Deficit (*n*, %)	0%	0%	NA
Postoperative Flexion Deficit (*n*, %)	0%	0%	NA
Postoperative Muscle Strength Deficit (*n*, %)	0%	0%	NA
Postoperative Apprehension test (*n*, %)	0%	0%	NA
Postoperative Positive J-sign (*n*, %)	1 (10%)	1 (5.3%)	0.579 ^3^
Hypoesthesia around the lower leg (*n*, %)	4 (40%)	10 (52.6%)	0.400 ^3^
Implant removal (*n*, %)	0%	0%	NA

^1^ Mann–Whitney U-test, ^2^ Wilcoxon Signed Rank Test, ^3^ Chi-square test. Abbreviations: Mg, Magnesium; Ti, Titanium; SD, Standard deviation; LKS, Lysholm Knee Score, NA: Not applicable.

## Data Availability

The original contributions presented in the study are included in the article, further inquiries can be directed to the corresponding author.

## Abbreviations

AHPLT	Anterior Half of the Peroneus Longus Tendon
ASA	American Society of Anesthesiologists
CT	Computed Tomography
MRI	Magnetic Resonance Imaging
MPFL	Medial Patellofemoral Ligament
MPFLR	Medial Patellofemoral Ligament Reconstruction
Mg	Magnesium
ROM	Range of Motion
SD	Standard Deviation
Ti	Titanium
TT	Tibial Tubercle
TG	Trochlear Groove
